# Sudden increase in human infection with avian influenza A(H7N9) virus in China, September–December 2016

**DOI:** 10.5365/WPSAR.2017.8.1.001

**Published:** 2017-01-18

**Authors:** Lei Zhou, Ruiqi Ren, Lei Yang, Changjun Bao, Jiabing Wu, Dayan Wang, Chao Li, Nijuan Xiang, Yali Wang, Dan Li, Haitian Sui, Yuelong Shu, Zijian Feng, Qun Li, Daxin Ni

**Affiliations:** a Public Health Emergency Center, Chinese Center for Disease Control and Prevention, Beijing, China.; b Chengdu Prefecture Center for Disease Control and Prevention, Sichuan, China.; c Jiangsu Provincial Center for Disease Control and Prevention, Jiangsu, China.; d Anhui Provincial Center for Disease Control and Prevention, Anhui, China.; e Institute for Viral Disease Control and Prevention, Chinese Center for Disease Control and Prevention, Beijing, China.; f Chinese Center for Disease Control and Prevention, Beijing, China.; * These authors contributed equally to this work.

## Abstract

Since the first outbreak of avian influenza A(H7N9) virus in humans was identified in 2013, there have been five seasonal epidemics observed in China. An earlier start and a steep increase in the number of humans infected with H7N9 virus was observed between September and December 2016, raising great public concern in domestic and international societies. The epidemiological characteristics of the recently reported confirmed H7N9 cases were analysed. The results suggested that although more cases were reported recently, most cases in the fifth epidemic were still highly sporadically distributed without any epidemiology links; the main characteristics remained unchanged and the genetic characteristics of virus strains that were isolated in this epidemic remained similar to earlier epidemics. Interventions included live poultry market closures in several cities that reported more H7N9 cases recently.

## Introduction

The first three H7N9 cases were reported in China on 31 March 2013. (*1*) Four epidemics have been observed in China between February 2013 and September 2016. Studies have shown that the demographic, epidemiologic and virologic characteristics of H7N9 cases from these epidemics remained unchanged, while the epidemic curve illustrated a decline in the magnitude of outbreaks over time, particularly in the third and fourth seasons in 2015 and 2016. (*2*, *3*) However, since September 2016, not only has the fifth outbreak started earlier than usual, but a steep increase in the number of humans infected with H7N9 virus has also been observed, causing domestic and international concern. On 9 January 2017, China notified the World Health Organization through the International Health Regulations mechanism of 106 cases. (*4*) An analysis of recently reported human cases with H7N9 was conducted to describe the epidemiological characteristics of the current epidemic. As the epidemic is ongoing, China remains vigilant and is monitoring outbreaks closely.

## Methods

### Surveillance system

The surveillance system and identification procedure for H7N9 infection has not changed in China since 2013. (*1*) A suspected H7N9 case in China is identified through the Chinese surveillance system for pneumonia of unknown etiology (PUE). In addition, suspected H7N9 cases with mild or moderate illness are identified from the Chinese sentinel surveillance system for influenza-like illness (ILI). The information from these systems are reported to the Internet-based National Notifiable Infectious Disease Report and Surveillance System (NNIDRSS). Each clinically diagnosed H7N9 case is confirmed by real-time reverse transcriptase polymerase chain reaction (RT–PCR), conventional RT–PCR, virus isolation, or a fourfold rise in H7N9 antibody titres in serology using laboratory methods and procedures as previously described. (*5*)

According to the national protocol of H7N9 disease control and prevention, (*6*) once a suspected H7N9 case is identified in one jurisdiction, the local Center for Disease Control and Prevention (CDC) conducts a field investigation, defines and monitors the close contacts for seven days, enhances ILI and PUE surveillance in medical institutions that are secondary level and above for two weeks, and collects environmental samples from possible exposure locations and tests them for H7N9 virus. The method of identification and follow-up of close contacts has been described previously. (*1*)

### Data collection

The epidemics were defined according to the seasonality of the disease. For comparability with a prior report, (*2*) we defined the epidemic duration from 1 September to 31 August of the following year, with the exception of the first epidemic. The first epidemic started on 19 February 2013. This date corresponds to the illness onset date of the first H7N9 case. Therefore, 1 September 2016 marks the beginning of the fifth season.

In this study, the demographic information of recent H7N9 cases, including age, sex, location of residence and occupation, were obtained from the NNIDRSS. The field epidemiological investigation reports were collected from local CDCs as a supplementary source to determine clinical severity and time interval between date of illness onset and date of first visit to clinic, first hospitalization, diagnosis and receiving oseltamivir treatment. The Protocol for Diagnosis and Treatment for Human Infection with A(H7N9) Influenza Virus (*6*, *7*) was followed to define a severe case as having any of the following: a chest X-ray indicative of multilobar lesions or a > 50% increase in the size of the lesions within a 48 hour period; dyspnea or a respiratory rate of greater than 24 times per minute for adults; severe hypoxia defined as less than or equal to 92% oxygen saturation while receiving 3–5 litres of supplemental oxygen per minute; or shock, acute respiratory distress syndrome or multiple organ dysfunction syndrome. Field investigation reports were reviewed to collect information regarding control measures and interventions implemented by local governments.

Detailed exposure information was abstracted from the field investigation reports. To assess exposure to live poultry markets (LPMs), we defined LPM as a market where live poultry is sold and could be touched. Individuals were considered to have a history of live poultry-related exposure in our analysis if they went to an LPM or bought poultry or other food in an LPM or touched poultry that was bought from an LPM within the past seven days. The definitions of other exposures, including direct and indirect contact with poultry, are previously described in a prior study conducted by China CDC. (*8*)

### Data analysis

To describe the epidemic, maps illustrating the geographic distribution of cases at provincial level were generated. Using the date of illness onset of confirmed H7N9 cases, an epidemic curve was generated to illustrate five epidemics between 2013 and 2016. A separate epidemic curve was created specifically for the 2016 epidemic. The number of reported cases, affected provinces, counties and newly affected counties were compared among the second, third, fourth and fifth epidemics. We also described the demographic and epidemiological characteristics of confirmed cases in the fifth epidemic and compared them to the earlier epidemics. For the purpose of this analysis, Hong Kong Special Administrative Region SAR (China), Macao SAR (China) and China, Taiwan, China were not included.

### Ethics approval and consent to participate

All the case information was collected according to the regulations of the Law of Communicable Diseases Prevention and Control of the People’s Republic of China as part of an emergency response; therefore, the study was exempt from obtaining ethics approval and participant consent.

## Results

As of 31 December 2016, a total of 889 confirmed H7N9 cases, including 361 deaths, were reported from 19 provinces in China since the first cases were reported in 2013. Nine provinces reported cases in all five epidemics (**Fig. 1a**). The H7N9 epidemics usually occurred within the winter–spring season except for the first epidemic in 2013. The outbreaks usually started in October, significantly increased in late December and then peaked in January of the next year (**Fig. 1b**). However, since September 2016, not only has the fifth epidemic begun earlier than usual, but a steep increase in the number of humans infected with H7N9 virus has also been observed in early December. As of 31 December 2016, 114 confirmed cases, including 42 deaths, have been reported from seven provinces, affecting 75 counties (**Fig. 1c**).

**Figure 1a:**
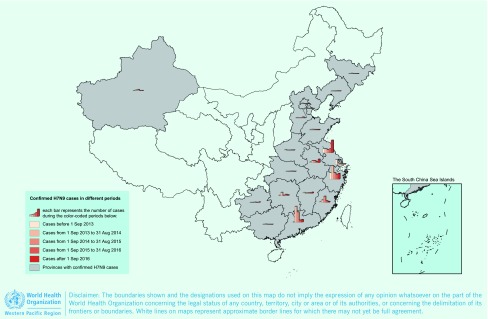
**Geographic distribution of human infection with H7N9 virus in China [excluding Hong Kong SAR (China), Macao SAR (China) and Taiwan, China], February 2013–December 2016**

**Figure 1b:**
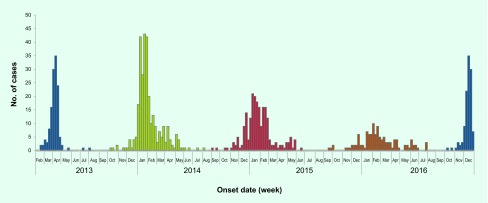
**Epidemic curve of human infection with H7N9 virus in China [excluding Hong Kong SAR (China), Macao SAR (China) and Taiwan, China] by week, February 2013–December 2016**

**Figure 1c:**
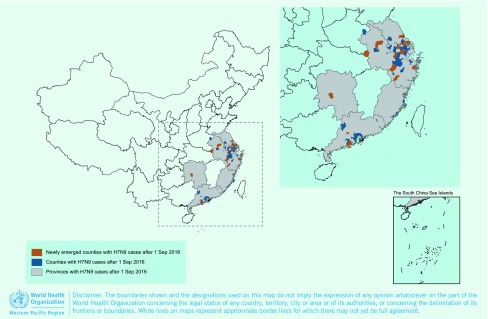
**Geographic distribution of human infection with H7N9 virus in China [excluding Hong Kong SAR (China), Macao SAR (China) and Taiwan, China], September 2016–December 2016**

The first case of the fifth epidemic had illness onset on 28 September 2016 in Zhejiang Province. In September, October and November 2016, a total of eight cases were reported in four provinces (Jiangsu, Zhejiang, Fujian, Guangdong), which is similar to the number of cases during the same period in prior epidemics. However, since 1 December 2016, the number of cases has substantially increased, with 106 cases reported in December 2016 alone (**Fig. 1d**). As of 31 December 2016, the number of reported cases in the fifth epidemic was 11.4, 2.7 and 6.1 times that observed in the corresponding periods in the second (10 cases), third (31 cases) and fourth (16 cases) epidemics, respectively.

**Figure 1d:**
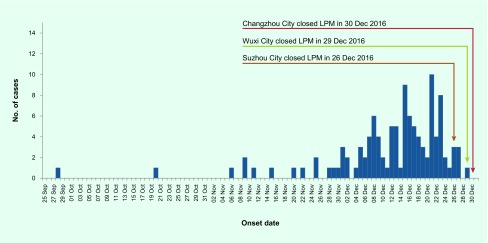
**Epidemic curve of human infection with H7N9 virus in China [excluding Hong Kong SAR (China), Macao SAR (China) and Taiwan, China] by day, September 2016–December 2016**

In the fifth epidemic, the number of cases were higher and the cases were more widespread than the second, third and fourth epidemics (**Table 1**). The number of provinces affected by the H7N9 virus in the fifth epidemic increased from four provinces (Jiangsu, Zhejiang, Fujian and Guangdong) in September, October and November 2016, to seven provinces (Jiangsu, Zhejiang, Anhui, Guangdong, Fujian, Hunan, Shanghai) by 31 December 2016. In the fifth epidemic, the number of newly affected counties, where no case was reported in prior epidemics, was 23, while the number of newly affected counties in the second, third and fourth epidemics was 4, 13 and 0, respectively (**Table 1**).

**Table 1:** Comparison of geographic distribution of human infection with H7N9 virus in the second, third, fourth and fifth epidemics*

**-**	Sep–Dec 2016	Sep–Dec 2015	Sep–Dec 2014	Sep–Dec 2013
No. of reported cases	114	16	31	10
No. of affected provinces	7	4	8	2
No. of affected counties	75	16	24	6
No. of newly affected counties	23	0	13	4

* The epidemic duration is defined as 1 September to 31 August in the next year. The first epidemic started on 19 February 2013, the date of the first H7N9 case illness onset. For comparison, only data of cases reported from 1 September to 31 December in each epidemic were selected.

Among the 114 cases reported to China CDC in the fifth epidemic, the median age was 55 years (range: 23–91); 68% were male (77/114); a quarter (29/114) were farmers, followed by retirees, persons who perform housework and persons who are unemployed. Of note, detailed clinical and exposure information within 10 days before illness onset was collected on 97 (85%) of the cases. All 97 cases developed pneumonia, and 87 (90%) of them had severe illness. Most (60/97, 62%) cases lived in urban areas, which remained similar to the earlier epidemics. (*2*) But in Zhejiang Province, most (16/21, 76%) cases lived in rural areas, which was higher than that in the prior epidemics (60%). Of the 97 cases with detailed exposure history, 87 (90%) reported exposure to live poultry, including LPMs (72/87 cases, 83%) and backyard poultry (10/87 cases, 11%) and 5 (6%) were themselves poultry workers. The proportion of cases with history of exposure to LPMs was higher in the current epidemic period than the 2013–2016 period (83% vs 69%) (**Table 2**).

**Table 2:** Comparison of demographic and epidemiological characteristics of H7N9 virus infections reported by time period, 19 February 2013–31 December 2016

Characteristics	H7N9 infections reported during Sep–Dec 2016 (*n* = 114)	H7N9 infections reported during Feb 2013–Aug 2016 (*n* = 775)2
Median age (range), years	55 (23–91yrs)	57 (9 mos-91yrs)
Male, *n* (%)	77 (68)	533 (69)
**Male age group, *n* (%)**		
0–14	0 (0)	21 (4)
15–29	1 (1)	27 (5)
30–44	11 (14)	84 (16)
45–59	31 (40)	159 (30)
60–74	23 (30)	163 (31)
> 75	11 (14)	79 (15)
**Female age group, *n* (%) **		
0–14	0 (0)	23 (10)
15–29	0 (0)	15 (6)
30–44	9 (24)	38 (16)
45–59	17 (46)	73 (30)
60–74	7 (19)	55 (23)
> 75	4 (11)	38 (16)
**Living area, *n* (%) **		
Cities and towns	60/97 (62)	438/775 (57)
Countryside and villages	37/97 (38)	337/775 (43)
**Occupation, *n* (%)**		
Farmer	29 (25)	210 (27)
Retiree	23 (20)	184 (24)
Person who does housework or is unemployed	22 (19)	91 (12)
Other occupations ^a^	40 (35)	290 (37)
**Live poultry-related exposure history, *n* (%)**	87/97 (90)	659 (85)
Exposed to LPM or poultry from LPM	72/87 (83)	457 (69)
Exposed to household poultry raised in backyard or neighbour's backyard	10/87 (11)	163 (25)
Occupational exposure ^b^	5/87 (6)	39 (6)
**Severe illness, *n* (%)**	87/97 (90)	506/592 (86)
Time interval, median days (IQR)	-	-
From onset to first visit to clinic	2 (1–3)	1 (0–4)
From onset to first hospitalization	4 (2–5)	4 (3–7)
From onset to diagnosis	9 (6–10)	8 (6–11)
From onset to start oseltamivir treatment	5 (4–6)	6 (4–8)

^a^ Other occupations include workers, cadres (persons working in government or government-affiliated institutions), business service providers, children, students, etc.

^b^ Occupational exposure refers to a person who raises, transports, sells, slaughters live poultry or does other jobs related to live poultry for a living.

In the current epidemic, the median time intervals between illness onset and initial medical consultation, hospitalization, diagnosis and time to antiviral treatment initiation were 2, 4, 9, 5 days, respectively; these remained similar to the earlier epidemics. Only 5% (3/58) of cases received oseltamivir within 48 hours of symptom onset in the current epidemic.

Two clusters, each cluster including two cases, were identified through close contact identification and follow-up and were reported from Jiangsu and Anhui provinces. Limited human-to-human transmission could not be ruled out in these two clusters. In Jiangsu cluster, the index case was a 66-year-old man, who had illness onset on 25 November 2016 and went to a hospital for outpatient treatment on 26 and 27 November. He was admitted to the hospital on 28 November. He was diagnosed on 4 December and died on 12 December. He had no underlying medical conditions; he had visited a LPM to buy food every day within 10 days before his illness onset. He had no direct contact with live poultry in the market. He lived alone, but after his hospitalization, his 39-year-old daughter, who had taken care of her father in hospital and had close contact with her father without personal protection for three days (28–30 November), became the second confirmed case. The onset of her illness was on 6 December. She was admitted to the hospital on 8 December and diagnosed on 15 December. She had no underlying medical conditions and had no live poultry or LPM exposure before the illness onset, except taking care of her father.

In the Anhui cluster, the index case was a 66-year-old man who developed fever and cough on 16 December 2016, and was admitted to the nephrology ward in the hospital on 17 December because of his diabetic nephropathy and hypertension. His condition deteriorated and he was transferred from the nephrology ward to the intensive care unit on 19 December. He was diagnosed on 19 December and died on 20 December. He lived alone and had visited an LPM to buy food every day within 10 days before his illness onset. He had no direct contact with live poultry in the market. The second case in this cluster was a 62-year-old man. He was admitted to the hospital for oedema. He and the index case stayed in the same room in the nephrology ward for approximately 20 hours. He had physical contact with the index case when assisting the index case to the bathroom. He had illness onset on 22 December and oseltamivir was given to him on the same day. He was diagnosed on 23 December. He had no history of exposure to live poultry or LPM before the illness onset.

During the fifth epidemic, as of 31 December 2016, a total of 33 H7N9 virus strains were isolated from 45 specimens collected from 40 confirmed cases in five provinces. All 33 viruses had completed full genetic analyses, and the genetic markers of mammalian adaptation and antiviral resistance of virus strains that were isolated in the fifth epidemics remained similar (Dr Yuelong Shu in China CDC, unpublished data) to earlier epidemics. (*9*) The genetic sequences of these viruses will be shared with the international community through the usual channels.

## Discussion

Our analysis showed that the current epidemic corresponding to the fifth H7N9 epidemic started in September and experienced a steep increase in early December. This indicates that the fifth epidemic began earlier than the epidemics in 2013–2015 that started in October, significantly increased in late December and reached their peaks in January of the following year. In the fifth epidemic, the number of cases seemed to increase more rapidly than was observed in prior epidemics. There were newly affected counties in the fifth epidemic in comparison with the earlier epidemics, indicating a geographic spreading of the virus. Except on two clusters, the cases had no epidemiological link, indicating human infection with H7N9 virus in China was still sporadic. Regardless, the demographic characteristics of cases, such as age and sex distribution and exposure history in the fifth epidemic, were similar to those in earlier epidemics. (*2*, *3*) Consistent with a prior report, (*10*) elderly people, especially those with underlying medical conditions, remain the most vulnerable population.

Live poultry exposure, especially LPM exposure, remained the major risk factor of infection. Previous studies determined that LPM exposure was associated with increased risk of infection with H7N9 virus. (*8*, *10*) The proportion of cases with history of LPM exposure was higher than that in earlier epidemics, indicating that LPM exposure remained the major risk factor of infection in the fifth epidemic. Control measures at LPMs had been determined to be effective to control H7N9 outbreaks. (*11*) To control the epidemic, strict market management measures, such as market closures, had been implemented by the local governments of severely affected jurisdictions such as Suzhou (from 26 December 2016), Wuxi (from 29 December 2016) and Changzhou (from 30 December 2016) in Jiangsu Province, (*12*-*14*) and Hefei (from 7 January 2017) in Anhui Province [Dr Jiabing Wu in Anhui CDC, personal communication]. While in Zhejiang and Guangdong provinces, (*15*, *16*) live poultry trade has been permanently prohibited in the main urban areas in all prefectures, and all live poultry slaughtering processes must be centralized. As the traditional Chinese New Year is approaching, the consumption of poultry among the general population will be increasing, which will pose higher risk to residents, especially in the areas where LPMs have not been closed. It is highly likely that sporadic cases will continue to be reported. Whenever influenza viruses are circulating in poultry, sporadic infections or small clusters of human cases are possible, especially in people exposed to infected poultry or contaminated environments.

There were two clusters reported in the fifth epidemic from Jiangsu and Anhui provinces, and limited human-to-human transmission between two individuals cannot be ruled out. Although the genetic markers of mammalian adaptation and antiviral resistance of virus strains that were isolated in the fifth epidemic remained similar to earlier epidemics. Continued monitoring of the virus and outbreaks is important as the pandemic potential of H7N9 remains.

There were some possible reasons for the sudden increase of H7N9 cases in the fifth epidemic. One is increased environmental contamination by the H7N9 virus. Environmental contamination has been determined as an alert for the emergence of H7N9 cases. (*17*, *18*) According to the routine environmental surveillance in affected provinces like Jiangsu, Zhejiang and Guangdong, the positive rate of environmental samples collected from LPMs or other live poultry-related environments increased in December 2016 and was higher compared to the relative periods of the earlier years (Dr Changjun Bao in Jiangsu CDC, unpublished data; Dr Enfu Chen in Zhejiang CDC, unpublished data; and Dr Min Kang in Guangdong CDC, unpublished data). Another possible reason is that it is simply an early epidemic of influenza disease. There were early increases of ILI reports in the prior influenza seasons (ILI surveillance weekly report by China CDC): about two months earlier for the southern provinces and one month earlier for the northern provinces.

In response to the epidemic situation, a series of control measures and interventions have already been implemented by national authorities. Before the beginning of the fifth epidemic, to enhance surveillance of avian influenza disease, China CDC set a monthly risk assessment mechanism with provincial CDCs that has been ongoing since August 2016. China National Health and Family Planning Commission (NHFPC) organized a multidepartment official supervision to six provinces in November 2016. China NHFPC conducted joint supervision with Ministry of Agriculture and Ministry of Industry and Commerce to Zhejiang, Jiangsu and Anhui provinces where the number of reported cases at the early stage of the fifth epidemic was higher than other provinces at the end of December 2016. China NHFPC organized a multidepartment joint technical meeting including Ministry of Agriculture and Ministry of Industry and Commerce to discuss control measures. China NHFPC and China CDC strengthened risk communication with the general public through a hotline, a web site, television and social chat applications such as WeChat (Tencent Holdings Limited). China CDC released a guideline to provincial CDCs to enhance H7N9 case detection and reporting, clinical management, nosocomial infection control, specimen collection and transportation, laboratory testing and virological analyses, field investigation and disease control in December 2016. The China Ministry of Agriculture issued a H7N9 virus elimination plan in 2014 to control infection and the spread of H7N9 virus among the poultry population, including control measures to decrease the exposure risk to residents during poultry raising, transportation and commercial trade. (*19*)

The study was unable to more thoroughly describe the fifth epidemic since the H7N9 outbreak is still ongoing. It was not possible to calculate the mortality rate as some cases are still receiving medical treatment in hospital. All close contacts during field investigation may not have been traced. Some cases may have been missed because the H7N9 cases were mainly identified through the PUE surveillance system, while some mild cases were identified by the ILI surveillance system. (*20*) Viruses have been isolated from specimens collected from 35% (40/114) of confirmed cases as of 11 January 2017. The laboratory testing is ongoing.

In conclusion, this study described the sudden increase in cases that occurred earlier than in previous years and that were mainly urban and significantly associated with exposure at LPMs Aside from two instances of possible human-to-human transmission between two individuals, cases remain sporadic in China.
